# Acute high‐protein feeding induces hepatic amino acid accumulation in diabetic male mice prior to transcriptional adaptation

**DOI:** 10.14814/phy2.71117

**Published:** 2026-09-25

**Authors:** Hiroaki Sumioki, Eisuke Tomatsu, Yusuke Seino, Shihomi Hidaka, Shinji Ueno, Sayumi Kanie, Koki Nishida, Takuya Haraguchi, Naoya Murao, Haruki Fujisawa, Takeshi Takayanagi, Yoshihisa Sugimura, Yoshitaka Hayashi, Atsushi Suzuki

**Affiliations:** ^1^ Department of Endocrinology, Diabetes and Metabolism Fujita Health University School of Medicine Toyoake Aichi Japan; ^2^ Yutaka Seino Distinguished Center for Diabetes Research, Kansai Electric Power Medical Research Institute Kyoto Japan; ^3^ Department of Endocrinology Research Institute of Environmental Medicine, Nagoya University Nagoya Aichi Japan; ^4^ Department of Endocrinology Nagoya University Graduate School of Medicine Nagoya Aichi Japan

**Keywords:** amino acid, glucagon, high‐protein diet, hyperglycemia, insulin, liver metabolism

## Abstract

Glucagon plays a pivotal role in hepatic amino acid metabolism; acute high‐protein diet feeding and diabetic condition both induce enhanced glucagon secretion. However, it remains unknown whether these two conditions alter hepatic amino acid metabolism through the same or different pathways. In this study, streptozotocin‐induced diabetic (STZ‐D) and nondiabetic (ND) mice received liquid normal control (LNC) or liquid high‐protein (LHP) diet. Plasma and hepatic parameters were analyzed 2 h post‐feeding. LHP increased plasma essential amino acids in both groups. Hepatic amino acid accumulation in LHP‐fed STZ‐D mice was increased compared to that in LNC‐fed STZ‐D mice but not in ND mice. In addition, LHP reduced hepatic glycogen while the expression of amino acid metabolism and urea cycle genes remained unchanged both in ND and STZ‐D mice. Compared with ND mice, STZ‐D mice showed upregulation of genes involved in gluconeogenesis and amino acid metabolism, together with an accumulation of early glycolytic intermediates and glycogen. These findings indicate that acute high‐protein intake induces non‐transcriptional regulation of amino acid metabolism within 2 h. The altered hepatic amino acid profile in STZ‐D mice is likely to be driven by increased hepatic amino acid uptake and accelerated gluconeogenesis.

## 1 INTRODUCTION

Oral glucose ingestion stimulates insulin secretion in both nondiabetic (ND) and type 2 diabetic (T2D) individuals; the early‐phase response is characteristically attenuated in T2D (Mitrakou et al., 1990; Yabe et al., 2015). Furthermore, while glucose suppresses plasma glucagon in individuals without T2D, it paradoxically leads to a slight elevation in those with T2D (Mitrakou et al., 1990; Yabe et al., 2015). In contrast, protein ingestion alone stimulates insulin secretion in both groups, albeit to a lesser extent than glucose (Alsalim et al., 2016; Ichikawa et al., 2023). Notably, protein serves as a potent secretagogue for glucagon regardless of glycemic status (Alsalim et al., 2016; Ichikawa et al., 2023). Consequently, mixed meals containing protein result in a concomitant rise in both hormones (Alsalim et al., 2016; El et al., 2021; Kondo‐Ando et al., 2019; Yabe et al., 2015).

During the postprandial state, insulin orchestrates glucose disposal through hepatic glycogen synthesis and peripheral glucose uptake (Yanagisawa, 2023). While insulin promotes amino acids uptake into peripheral tissues for protein synthesis, glucagon contributes to amino acid metabolism by stimulating hepatic gluconeogenesis, thereby facilitating the conversion of amino acids into glucose (James et al., 2017), indicating that these two hormones participate in amino acid homeostasis.

In type 1 diabetic (T1D) and T2D individuals, impaired insulin secretion and action combined with paradoxical hyperglucagonemia shifts the insulin‐to‐glucagon ratio (IGR) toward glucagon dominance (Dao et al., 2025; Ichikawa et al., 2019; Jiang & Zhang, 2003; Mitrakou et al., 1990; Müller et al., 1970; Wewer Albrechtsen et al., 2016; Yabe et al., 2015). This altered hormonal balance likely affects postprandial amino acid profiles, supporting findings that lean T2D individuals exhibit elevated plasma amino acid levels compared with nondiabetic controls (Alqudah et al., 2021; Grøndahl et al., 2024). While the increase in plasma amino acid concentrations following oral amino acid mixture ingestion during a euglycemic clamp was similar between individuals with T1D and ND (Rossetti et al., 2008), some of the plasma amino acid levels were higher in T1D individuals (Kawamori et al., 2023).

As glucagon and insulin coordinately regulate hepatic amino acid and glucose metabolism (James et al., 2017; Janah et al., 2019; Petersen & Shulman, 2018), we hypothesized that acute high‐protein diet feeding would similarly alter IGR due to its robust induction of glucagon secretion. However, it remains unclear whether these two conditions alter hepatic amino acid metabolism through the same or different pathways. We therefore compared the metabolic responses to an acute high protein diet feeding in diabetic and nondiabetic states in streptozotocin (STZ)‐induced diabetic (STZ‐D) and ND mice fed with a liquid high‐protein (LHP) diet or a liquid normal control (LNC) diet.

## 2 MATERIALS AND METHODS

### 2.1 Experimental animals and diets

Eight‐week‐old male C57BL/6J wild‐type mice (CLEA Japan, Inc., Tokyo, Japan) were maintained under a 12:12‐h light:dark cycle with ad libitum access to a standard laboratory chow diet (CLEA Rodent Diet CE‐2; CLEA Japan, Inc., Tokyo, Japan) and water. Mice were assigned to two groups: ND and STZ‐D mice. To induce diabetes, mice received intraperitoneal injections of STZ (50 mg/kg; Sigma‐Aldrich, Merck KGaA, Darmstadt, Germany; Cat. No. S0130) for five consecutive days as previously described in detail (Iida et al., 2016). Control mice were injected with saline for five consecutive days. After an 11‐day interval from the final injection, the mice were fasted for 16 h and then administered a liquid diet at 13 μL/g body weight (BW). The LNC group received a diet adjusted to 67.0% carbohydrate, 14.6% protein, and 18.4% fat (2.19 kcal/mL) (based on Meiji Maybalance Reha Support Mini; Meiji Co., Ltd., Tokyo, Japan). The LHP group received a diet supplemented with SAVAS protein (Meiji Co., Ltd.), resulting in a final composition of 23.4% carbohydrate, 58.2% protein, and 18.4% fat (2.18 kcal/mL). All the mouse experiments were performed in accordance with Fujita Health University protocols. The animal study protocol was approved by the Ethics Committee of Fujita Health University (AP18002: approval date 14 May 2018).

### 2.2 Plasma biochemical analyses

Blood was collected from the tip of the tails, and blood glucose levels were measured with an Antsense Duo Small Electrode Glucose Analyzer (Horiba, Kyoto, Japan). Blood samples were centrifuged (2000 g, 10 min, 4°C) twice, and the collected plasma samples were stored at −80°C until the biochemical analyses. Plasma hormone levels were measured using the following assays: insulin, Mouse/Rat Insulin ELISA Kit (Morinaga Institute of Biological Science, Kanagawa, Japan; Cat. No. M1108); Fibroblast Growth Factor 21 (FGF21), Mouse and Rat FGF21 ELISA Kit (BioVendor Inc., Brno, Czech Republic; Cat. No. RD291108200R); Glucagon ELISA Kit (Mercodia, Uppsala, Sweden; Cat. No. 10‐1281‐01); Glucose‐dependent insulinotropic polypeptide (GIP), GIP (total) ELISA kit (Millipore, Billerica, MA, USA; Cat. No. EZRMGIP‐55K); and corticosterone, Corticosterone Enzyme Immunoassay Kit (Arbor Assays, Ann Arbor, MI, USA; Cat. No. K014‐H1). Hormone concentrations were measured once for each sample using the indicated assay kits, in accordance with the manufacturers' protocols. Plasma amino acid concentrations were measured by standard assays at SRL, Inc. (Tokyo, Japan), as previously described (Ueno et al., 2022; Watanabe et al., 2012). The insulin‐to‐glucagon ratio (IGR) was calculated by [plasma insulin (pmol/L)/plasma glucagon (pmol/L)] and homeostasis model assessment of insulin resistance (HOMA‐IR) was calculated by [fasting glucose (mg/dL) × fasting insulin (μU/mL)]/405 (Maekawa et al., 2018; Matthews et al., 1985).

### 2.3 Isolation of RNA and quantitative PCR

Mice were euthanized by inhalation of 5% isoflurane (MSD Animal Health Tokyo Japan) and sacrificed 2 h after feeding and liver samples were collected. Total Ribonucleic acid (RNA) was extracted, Complementary Deoxyribonucleic acid (cDNA) was synthesized, and quantitative polymerase chain reactions (qPCR) were performed using QuantStudio 7 Flex Real‐Time PCR System (Applied Biosystems, Thermo Fisher Scientific, Waltham, MA, USA; Cat. No. 4485688) as previously described (Nishida et al., 2024; Ueno et al., 2022). Target gene messenger RNA (mRNA) levels were normalized to β‐actin as an internal control and expressed as fold change relative to LNC‐fed ND mice. The sequence of primers and abbreviation of the gene names are summarized in Table 1.

**TABLE 1:** Primers used for quantitative real‐time PCR (qPCR).

Gene	Forward primers (5′–3′)	Reverse primers (3′–5′)
Phosphoenolpyruvate carboxykinase (*Pepck*)	GTGTTTGTAGGAGCAGCCATGAG	TAGCCGAAGAAGGGTCGCAT
Glucose‐6‐phosphatase, catalytic subunit (*G6pc*)	CGGATCTACCTTGCTGCTCA	AACAAGAAGATGGTGATGAGACAAT
Glucokinase (*Gck*)	AGACGAAACACCAGATGTATTCC	GAAGCCCTTGGTCCAGTTGAG
Glycogen synthase 2 (*Gys2*)	GGAAGAAACTCTATGACGGGTTATT	TCATCGATCATATTGTGAGTGGTC
Glycogen phosphorylase, liver form (*Pygl*)	GCACTACTACGACAAGTGTCCC	TAAATGGCCTCATCGCAG
Glutamic‐oxaloacetic transaminase (*Got*)	TTGGTCTCACATCACTGAGCA	GATGGAGGTAGCGACGTAATCTAG
Serine dehydratase (*Sds*)	CAGCTTCCATGCTGCCATCAAG	CCTCCTGGTCTGAGATGACCTC
Glutamic‐pyruvic transaminase (*Gpt*)	TCCAGGCTTCAAGGAATGGAC	CAAGGCACGTTGCACGATG
Glutamic‐pyruvic transaminase 2 (*Gpt2*)	AACCATTCACTGAGGTAATCCGA	GGGCTGTTTAGTAGGTTTGGGTA
Glutaminase 2 (*Gls2*)	CGTCCGGTACTACCTCGGT	TGTCCCTCTGCAATAGTGTAGAA
Alanine‐glyoxylate aminotransferase (*Agxt*)	ACCTGCAGGAGATGGGCTTA	GCACATAGCTGACGATGTCC
Argininosuccinate synthase 1 (*Ass1*)	GCGACTATGAGCCCATCGAC	GGCCCGCTCCTCTTTGTCAG
Arginase 1 (*Arg1*)	TTCTGGGAGGCCTATCTTACAGA	CCACTGCCGTGTTCACAGTA
Ornithine aminotransferase (*Oat*)	TCAGTGAGAGGGAAAGGGTT	CCGGATCTCATCCTCCTTGA
Carbamoyl‐phosphate synthase 1 (*Cps1*)	GGAGTGGATACAAGAATGCTGAC	GCAGGCGGATGACATTGTTTTT
Peroxisome proliferator‐activated receptor alpha (*Pparα*)	CCTGAACATCGAGTGTCGAATAT	GTTCTTCTTCTGAATCTTGCAGCT
Beta‐Actin (*β‐actin*)	CATCCGTAAAGACCTCTATGCCAAC	ATGGAGCCACCGATCCACA

### 2.4 Hepatic immunoblotting analysis

Liver proteins were extracted in radioimmunoprecipitation assay (RIPA) buffer containing PhosSTOP phosphatase inhibitor cocktail (Roche Diagnostics, Mannheim, Germany; Cat. No. 04906837001) and cOmplete Mini protease inhibitors (Roche Diagnostics; Cat. No. 11836170001) Total protein (30 μg) was separated on a 10% sodium dodecyl sulfate–polyacrylamide gel and transferred onto polyvinylidene difluoride membranes (Merck Millipore, Billerica, MA, USA; Cat. No. IPVH00010). After blocking with 3% bovine serum albumin in Tris‐buffered saline containing 0.1% Tween 20 (BSA/TBS‐T), the membranes were incubated with primary antibodies against phospho‐protein kinase B (p‐Akt; Ser473; Cat. No. 4060; 1:2000), Akt (Cat. No. 9272, 1:1000), phospho‐glycogen synthase kinase 3β (p‐GSK3β; Ser9; Cat. No. 9336; 1:1000), GSK3β (Cat. No. 12456, 1:1000), phospho‐cAMP response element‐binding protein (p‐CREB; Ser133; Cat. No. 9198; 1:1000), CREB (Cat. No. 9197, 1:1000), and glyceraldehyde‐3‐phosphate dehydrogenase (GAPDH) (Cat. No. 2118; 1:2000). All primary antibodies were obtained from Cell Signaling Technology (Danvers, MA, USA). Blots were imaged and quantified using ImageJ software (ver. 1.53e, National Institutes of Health, Bethesda, MD, USA) as previously reported (Nishida et al., 2024). The phosphorylation levels of AKT, CREB, and GSK3β were expressed relative to their corresponding total protein levels and normalized to the mean value of the ND‐LNC group.

### 2.5 Measurement of liver glycogen content

Liver samples (10 mg) were homogenized in 200 μL ddH2O, boiled for 10 min, and centrifuged (17,700 *g*, 10 min). The supernatant was analyzed using a Glycogen Colorimetric Assay Kit II (Abcam, Cambridge, UK; Cat. No. ab169558). Hepatic glycogen contents were measured once for each sample using the indicated assay kits, in accordance with the manufacturers' protocols.

### 2.6 Hepatic metabolome analysis

Metabolomic analysis was performed with modifications as previously described (Murao et al., 2025). Briefly, the liver tissue sample (30 mg) was homogenized with ice‐cold extraction buffer (41.9% methanol, 41.9% chloroform, and 16.2% ultrapure water containing the internal standard solution), deproteinized by ultrafiltration (Human Metabolome Technologies, Yamagata, Japan; Cat. No. UFC3LCCNB‐HMT), and lyophilized. Subsequently, metabolite concentrations were measured in both positive and negative ion modes using a G7100A capillary electrophoresis system coupled to a G6224A time‐of‐flight LC/MS (Agilent Technologies, Santa Clara, CA, USA). Chromatograms and mass spectra were analyzed using MassHunter Qualitative Analysis version 10.0 s (Agilent Technologies; RRID:SCR_015040, CA, USA). Annotation and quantification of chromatogram peaks were performed using a standard mixture (Human Metabolome Technologies, Yamagata, Japan; Cat. No. H3304‐1031, H3304‐1032, H3304‐1034, H3304‐1036).

### 2.7 Statistical analysis

Sample sizes were estimated from the expected effect size based on preliminary experimental trials. No randomization or blinding was used. Data are expressed as mean ± standard error of the mean (SEM). Statistical analyses were performed using two‐way ANOVA followed by Tukey's multiple comparisons test. A *p* value of <0.05 was considered statistically significant. All analyses were conducted using GraphPad Prism 10 for Windows (GraphPad Software, San Diego, CA, USA).

## 3 RESULTS

### 3.1 Metabolic and hormonal responses to LNC and LHP in ND and STZ‐D mice

We previously reported that mice treated with STZ (50 mg/kg) for 5 days showed hyperglycemia due to decreased insulin secretion from 1 week after post‐treatment compared with control mice (Iida et al., 2016). In the present study, we confirmed that STZ‐D mice exhibited significantly higher blood glucose levels and lower body weight than ND mice under fed ad libitum condition (Figure S1A,B).

**FIGURE 1:**
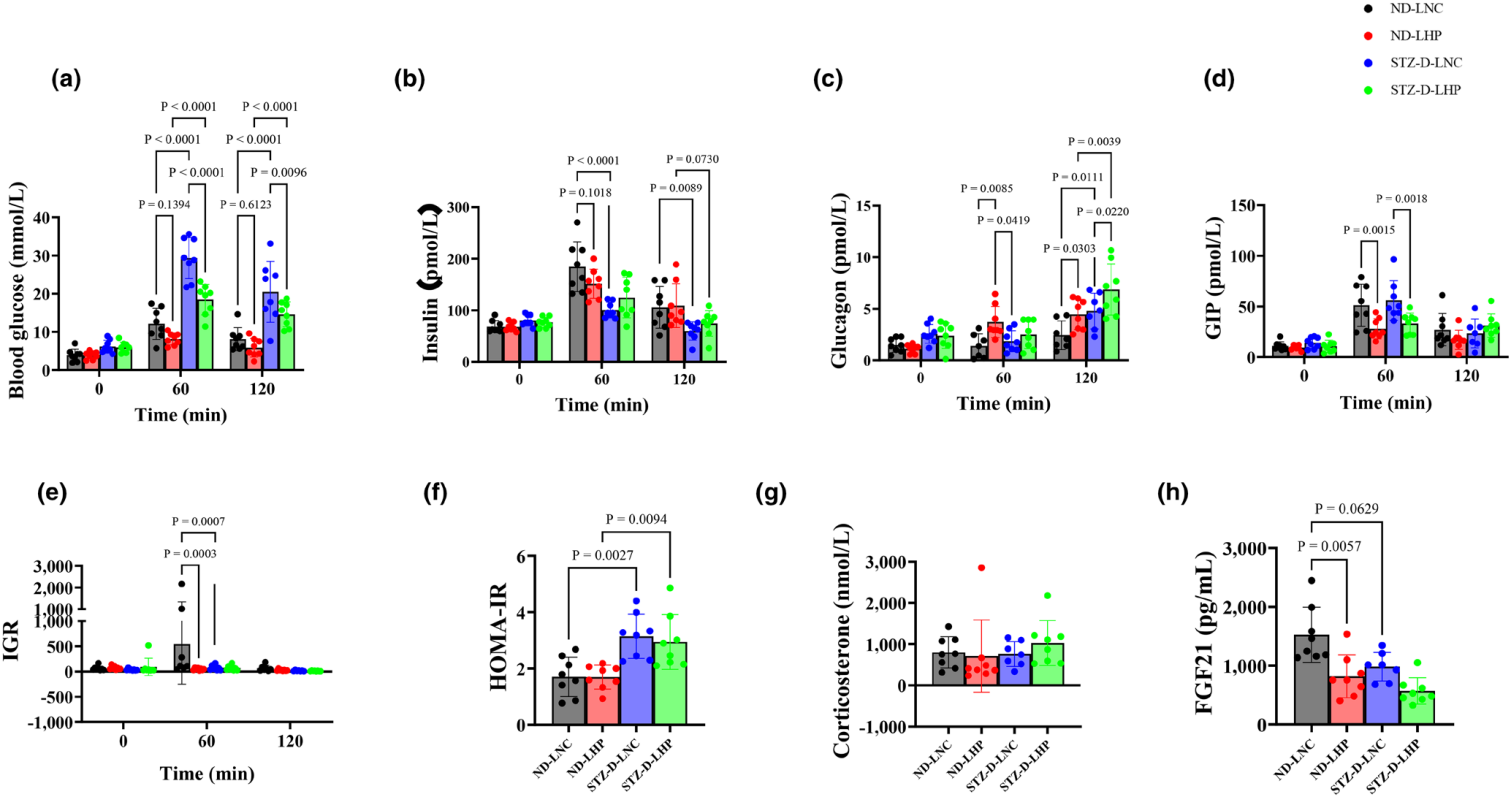
Effects of acute LHP administration on postprandial metabolic parameters in ND and STZ‐D mice. (a) Blood glucose levels; (b) plasma insulin concentrations; (c) plasma glucagon concentrations; (d) plasma GIP concentrations; (e) IGR; (f) HOMA‐IR; (g) plasma corticosterone levels and (h) plasma FGF21 concentrations at 120 min; black bars, ND mice fed LNC; red bars, ND mice fed LHP; blue bars, STZ‐D mice fed LNC; green bars, STZ‐D mice fed LHP; Data are presented as mean ± standard deviation (SD), *n* = 7–8 per group. Statistical analysis was performed using (a)–(h) two‐way ANOVA followed by Tukey's multiple comparison test. *p* Values are shown in the figure. FGF21, fibroblast growth factor 21; GIP, glucose‐dependent insulinotropic polypeptide; HOMA‐IR, homeostasis model assessment of insulin resistance; IGR, insulin‐to‐glucagon ratio; LNC, liquid normal diet; LHP, liquid high‐protein diet; ND, nondiabetic; STZ‐D, streptozotocin‐induced diabetic.

Postprandial blood glucose levels were higher in STZ‐D mice compared to those in ND mice under both diets; LHP administration effectively suppressed this elevation regardless of the diabetic condition (Figure 1a). The plasma concentrations of insulin and glucagon were increased under both LNC and LHP, with insulin concentration being higher at 60 min than at 120 min, whereas glucagon concentration was higher at 120 min than at 60 min. The insulin secretion in response to LNC was significantly attenuated in STZ‐D mice, whereas the response to LHP was comparable between ND and STZ‐D mice. Glucagon secretion was greater under LHP than under LNC in both ND and STZ‐D mice (Figure 1b,c). Regardless of the diabetic state, plasma GIP levels at 60 min were significantly lower in the LHP group than in the LNC group (Figure 1d). In ND mice, the insulin‐to‐glucagon ratio (IGR) at 60 min was significantly lower in the LHP group compared to that in the LNC group. In the LNC‐fed groups, the IGR at 60 min was significantly lower in STZ‐D mice than that in ND mice. Notably, in STZ‐D mice, no significant difference in IGR was observed between the LNC and LHP groups (Figure 1e). STZ‐D mice tended to have higher glucose and insulin levels before liquid food loading, although the difference was not statistically significant. However, STZ‐D mice showed significantly higher HOMA‐IR values than ND mice (Figure 1f). Thus, STZ‐D mice exhibited hepatic insulin resistance combined with attenuated insulin secretion in response to food loading. No significant differences in plasma corticosterone levels at 120 min were observed between the LNC and LHP diets in either ND or STZ‐D mice (Figure 1g). In ND mice, plasma FGF21 levels at 120 min were significantly lower in the LHP group than in the LNC group. Furthermore, LNC‐fed STZ‐D mice tended to exhibit significantly lower 120‐min FGF21 levels compared to LNC‐fed ND mice, but any difference did not reach the statistical significance. While FGF21 levels in STZ‐D mice exhibited a downward trend in the LHP group relative to the LNC group, there was not a statistically significant difference (Figure 1h).

### 3.2 Plasma amino acid profiles in ND and STZ‐D mice fed LNC or LHP

LHP administration significantly elevated the plasma concentrations of all essential amino acids (EAAs), including branched‐chain amino acids (BCAAs), compared to LNC regardless of diabetic status, except for phenylalanine in ND mice. On LNC ingestion, STZ‐D mice generally exhibited lower levels of amino acids than ND mice, except for arginine, aspartic acid, tryptophan, and BCAAs. On LHP ingestion, the concentrations of arginine, tyrosine, phenylalanine, lysine, tryptophan, and BCAAs were higher in STZ‐D mice. Among these, arginine, tyrosine, phenylalanine, tryptophan, and isoleucine concentrations were significantly increased in STZ‐D mice compared to ND mice (Table 2).

**TABLE 2:** Plasma amino acid concentrations in nondiabetic (ND) and streptozotocin‐induced diabetic (STZ‐D) mice fed a liquid normal control diet (LNC) or a liquid high‐protein diet (LHP) at 120 min post administration.

	Plasma amino acids	Mean ± SD				*p* Value			
		ND‐LNC	ND‐LHP	STZ‐D‐LNC	STZ‐D‐LHP	ND‐LNC vs. ND‐LHP	STZ‐D‐LNC vs. STZ‐D‐LHP	ND‐LNC vs. STZ‐D‐LNC	ND‐LHP vs. STZ‐D‐LHP
	(nmol/mL)								
NEAAs	Alanine	416.8 ± 74.0	470.5 ± 77.1	309.0 ± 61.6	442.9 ± 52.3	0.3908	0.0031	0.0188	0.8390
	Arginine	59.5 ± 10.3	67.8 ± 9.6	67.1 ± 9.0	82.0 ± 13.7	0.3593	0.0304	0.4429	0.0424
	Asparagine	48.3 ± 4.1	76.9 ± 14.3	37.0 ± 6.1	59.3 ± 7.9	<0.0001	0.0001	0.0576	0.0019
	Aspartic acid	12.0 ± 3.8	13.3 ± 4.4	15.0 ± 14.7	12.9 ± 4.3	0.9897	0.9554	0.8872	0.9997
	Cystine	5.4 ± 1.4	6.6 ± 1.7	5.2 ± 0.9	5.0 ± 0.5	0.6768	0.9983	0.9117	0.2115
	Glutamine	663.3 ± 48.5	749.5 ± 108.2	568.8 ± 100.1	675.6 ± 86.8	0.1999	0.0810	0.1417	0.3189
	Glutamic acid	44.4 ± 10.6	45.1 ± 8.9	42.9 ± 26.2	39.4 ± 8.8	0.9997	0.9722	0.9978	0.8929
	Glycine	204.0 ± 35.0	182.4 ± 15.8	155.9 ± 25.9	144.1 ± 12.9	0.3494	0.7931	0.0056	0.0317
	Proline	82.3 ± 9.2	135.8 ± 23.7	60.9 ± 10.1	118.9 ± 16.0	<0.0001	<0.0001	0.0174	0.0757
	Serine	125.6 ± 16.5	155.6 ± 22.5	93.3 ± 21.8	124.8 ± 14.6	0.0177	0.0123	0.0099	0.0144
	Tyrosine	50.2 ± 9.7	57.7 ± 10.5	48.0 ± 6.3	82.2 ± 32.7	0.8249	0.0036	0.9934	0.0441
EAAs	Phenylalanine	83.9 ± 8.1	86.2 ± 11.5	83.0 ± 10.2	107.6 ± 14.1	0.9722	0.0008	0.9984	0.0032
	Lysine	248.4 ± 28.6	377.0 ± 57.4	197.3 ± 34.8	400.9 ± 102.8	0.0012	<0.0001	0.3138	0.8407
	Methionine	68.6 ± 7.7	93.7 ± 14.0	52.0 ± 8.9	81.6 ± 12.3	0.0004	<0.0001	0.0192	0.1167
	Threonine	245.9 ± 29.5	487.1 ± 75.4	180.0 ± 48.2	359.0 ± 37.3	<0.0001	<0.0001	0.0534	0.0001
	Tryptophan	53.2 ± 7.4	85.3 ± 8.7	54.2 ± 10.4	96.5 ± 9.9	<0.0001	<0.0001	0.9936	0.0443
	Histidine	67.0 ± 5.3	79.9 ± 9.7	56.3 ± 6.0	74.7 ± 12.2	0.0170	0.0006	0.0556	0.5610
BCAAs	Isoleucine	91.8 ± 11.5	222.8 ± 61.0	105.8 ± 20.4	296.6 ± 85.4	0.0003	<0.0001	0.9498	0.0480
	Leucine	190.3 ± 34.2	457.9 ± 99.8	195.0 ± 34.9	568.3 ± 137.7	<0.0001	<0.0001	0.9995	0.0753
	Valine	192.0 ± 43.3	546.0 ± 99.9	202.4 ± 42.4	645.0 ± 178.9	<0.0001	<0.0001	0.9960	0.1809

*Note*: Data are presented as mean ± standard deviation (SD), *n* = 8 per group. Statistical analysis was performed using two‐way ANOVA followed by Tukey's multiple comparison test.

Abbreviations: BCAAs, branched‐chain amino acids; EAAs, essential amino acids; LHP, liquid high‐protein diet; LNC, liquid normal control diet; ND, nondiabetic; NEAAs, nonessential amino acids.

### 3.3 Hepatic amino acid profiles in ND and STZ‐D mice fed LNC or LHP

Next, we examined metabolite profiles in the liver. In ND mice, amino acid content did not differ significantly between the LNC and LHP groups, except for significantly higher glutamine content in the LHP group. In contrast, in STZ‐D mice, the LHP group showed significant increases in alanine, aspartic acid, proline, threonine, and BCAAs, along with a trend toward increased lysine levels; notably, glutamine was the sole exception, being significantly lower than in the LNC group. Furthermore, STZ‐D mice displayed significantly higher levels of asparagine, glycine, tyrosine, and phenylalanine, and a higher trend of methionine, compared to ND mice. These differences were further accentuated by LHP loading, which resulted in an increase in STZ‐D mice in all EAAs except for histidine (Table 3). The lower plasma amino acid levels and higher hepatic amino acid profiles suggested that amino acid uptake by the liver is accelerated in STZ‐D mice administered LHP.

**TABLE 3:** Hepatic amino acid in nondiabetic (ND) and diabetic (STZ‐D) mice fed a liquid normal control diet (LNC) or a liquid high‐protein diet (LHP) at 120 min post administration.

	Metabolite	Mean ± SD				*p* Value			
		ND‐LNC	ND‐LHP	STZ‐D‐LNC	STZ‐D‐LHP	ND‐LNC vs. ND‐LHP	STZ‐D‐LNC vs. STZ‐D‐LHP	ND‐LNC vs. STZ‐D‐LNC	ND‐LHP vs. STZ‐D‐LHP
	(pmol/mg tissue)								
NEAAs	Alanine	2372.6 ± 514.4	2199.6 ± 459.3	2553.3 ± 416.2	3670.2 ± 996.1	0.9469	0.0102	0.9403	0.0008
	Arginine	5.1 ± 1.6	4.0 ± 1.1	4.6 ± 1.0	5.8 ± 1.3	0.1792	0.1541	0.7924	0.0132
	Asparagine	89.8 ± 14.5	98.5 ± 19.4	160.9 ± 40.5	157.0 ± 28.0	0.9314	0.9929	0.0004	0.0031
	Aspartic acid	396.5 ± 101.8	381.6 ± 84.3	361.6 ± 45.7	598.1 ± 228.0	0.9962	0.0115	0.9553	0.0220
	Cystine	14.2 ± 7.4	13.8 ± 6.4	7.3 ± 2.1	9.5 ± 4.6	0.9976	0.7303	0.0199	0.2214
	Glutamine	3322.8 ± 635.2	4001.4 ± 517.6	3661.3 ± 441.2	2563.2 ± 380.5	0.0476	0.0009	0.5119	<0.0001
	Glutamic acid	1692.4 ± 532.8	1393.4 ± 521.7	1186.6 ± 163.8	1743.8 ± 627.4	0.6028	0.1228	0.1807	0.4746
	Glycine	1892.2 ± 254.5	2147.0 ± 216.9	2335.6 ± 259.4	2066.6 ± 158.9	0.1287	0.1012	0.0033	0.8849
	Proline	212.7 ± 55.5	160.4 ± 43.7	232.9 ± 59.1	393.9 ± 146.3	0.5914	0.0041	0.9605	<0.0001
	Serine	362.0 ± 64.6	396.3 ± 116.9	525.6 ± 130.2	649.6 ± 219.2	0.9630	0.3328	0.1340	0.0097
	Tyrosine	110.9 ± 23.6	132.4 ± 34.3	204.6 ± 50.0	246.2 ± 72.0	0.8189	0.3543	0.0054	0.0008
EAAs	Phenylalanine	102.4 ± 19.6	120.9 ± 27.2	178.0 ± 38.2	182.7 ± 43.4	0.7194	0.9932	0.0016	0.0101
	Lysine	587.8 ± 239.6	501.1 ± 114.7	702.5 ± 149.1	1457.6 ± 1091.7	0.9889	0.0555	0.9752	0.0114
	Methionine	48.3 ± 7.2	55.0 ± 15.1	78.4 ± 26.2	94.3 ± 30.6	0.9364	0.5308	0.0725	0.0135
	Threonine	528.6 ± 104.4	381.6 ± 96.2	522.8 ± 71.2	759.6 ± 188.8	0.1109	0.0048	0.9997	<0.0001
	Tryptophan	28.6 ± 6.2	24.7 ± 4.7	34.1 ± 7.4	36.5 ± 5.4	0.5410	0.8456	0.2465	0.0027
	Histidine	439.7 ± 87.4	451.4 ± 80.5	442.7 ± 46.5	513.5 ± 101.2	0.9900	0.2860	0.9998	0.3960
BCAAs	Isoleucine	208.6 ± 60.9	155.5 ± 33.4	224.0 ± 63.6	369.2 ± 117.4	0.4586	0.0028	0.9719	<0.0001
	Leucine	437.7 ± 125.8	340.0 ± 71.3	446.9 ± 113.3	754.7 ± 258.2	0.5677	0.0025	0.9993	<0.0001
	Valine	447.8 ± 102.4	288.9 ± 53.9	418.1 ± 103.2	771.4 ± 284.3	0.1776	0.0006	0.9781	<0.0001

*Note*: Data are presented as mean ± standard deviation (SD), *n* = 8 per group. Statistical analysis was performed using two‐way ANOVA followed by Tukey's multiple comparison test.

Abbreviations: BCAAs, branched‐chain amino acids; EAAs, essential amino acids; LHP, liquid high‐protein diet; LNC, liquid normal control diet; ND, nondiabetic; NEAAs, nonessential amino acids.

### 3.4 Hepatic glycogen content in ND and STZ‐D mice fed LNC or LHP

**FIGURE 2:**
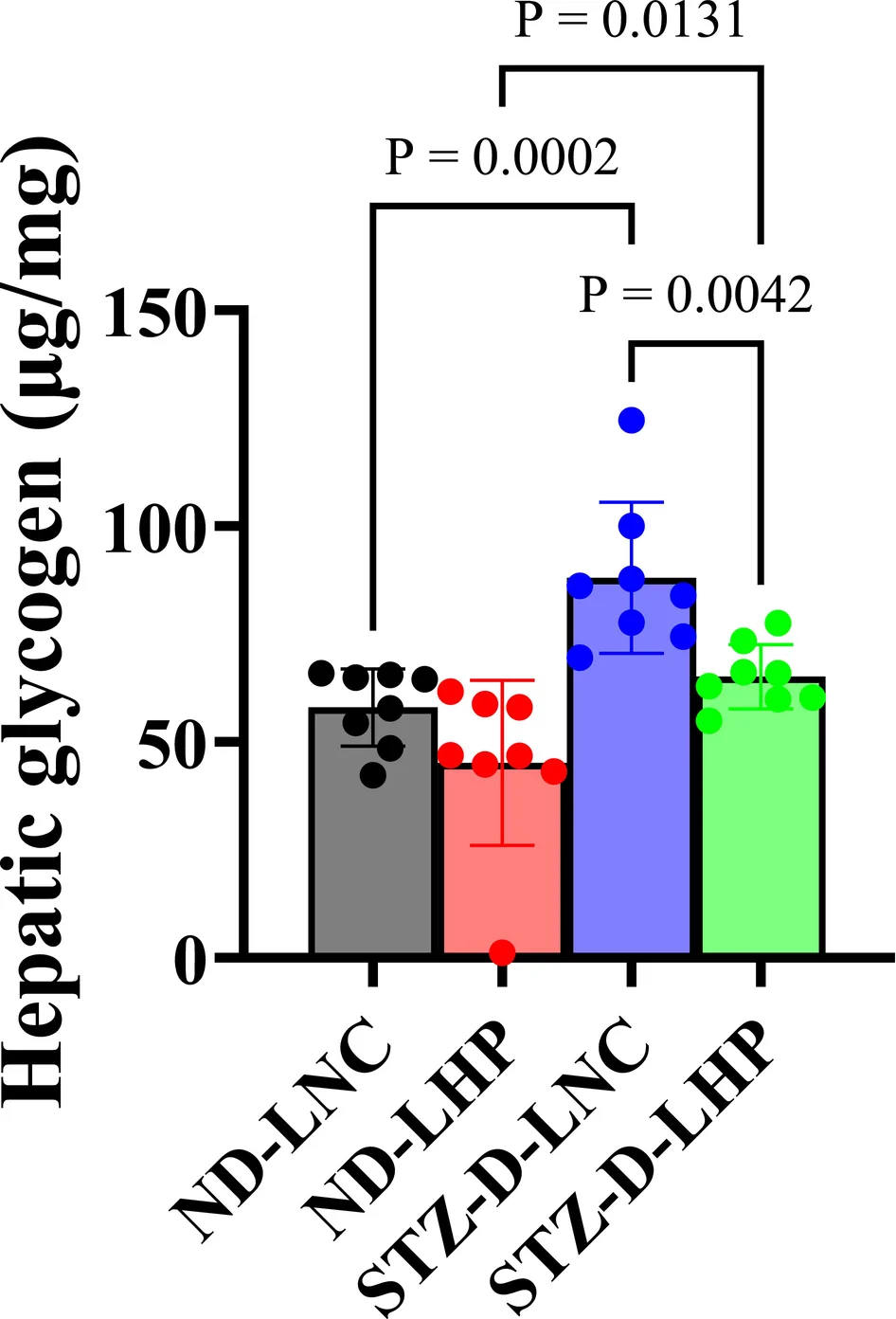
Hepatic glycogen content in ND and STZ‐D mice following acute LHP administration. Hepatic glycogen content was measured 120 min after loading with LNC or LHP in ND and STZ‐D mice; black bars, ND mice fed LNC; red bars, ND mice fed LHP; blue bars, STZ‐D mice fed LNC; green bars, STZ‐D mice fed LHP; Data are presented as mean ± standard deviation (SD), *n* = 8 per group. Statistical analysis was performed using two‐way ANOVA followed by Tukey's multiple comparison test. *p* Values are shown in the figure. LHP, liquid high‐protein diet; LNC, liquid normal diet; ND, nondiabetic; STZ‐D, streptozotocin‐induced diabetic.

Hepatic glycogen content was higher in STZ‐D mice than in ND mice, regardless of the dietary composition. Furthermore, in STZ‐D mice, the LHP group showed significantly lower glycogen content compared to the LNC group, while a similar downward trend was observed in ND mice (Figure 2).

### 3.5 Hepatic glycolytic intermediates in ND and STZ‐D mice fed LNC or LHP

In contrast, glycolytic metabolite levels showed no significant differences between the LNC and LHP groups in either ND or STZ‐D mice. However, STZ‐D mice exhibited significantly higher concentrations of the initial glycolytic metabolites: glucose 1‐phosphate, glucose 6‐phosphate, and fructose 6‐phosphate, compared to ND mice (Table 4).

**TABLE 4:** Hepatic glycolytic intermediate levels in nondiabetic (ND) and diabetic (STZ‐D) mice fed a liquid normal control diet (LNC) or a liquid high‐protein diet (LHP) at 120 min post administration.

Metabolite	Mean ± SD				*p* Value			
	ND‐LNC	ND‐LHP	STZ‐D‐LNC	STZ‐D‐LHP	ND‐LNC vs. ND‐LHP	STZ‐D‐LNC vs. STZ‐D‐LHP	ND‐LNC vs. STZ‐D‐LNC	ND‐LHP vs. STZ‐D‐LHP
(pmol/mg tissue)								
Glucose 1‐phosphate	64.9 ± 15.9	84.9 ± 20.9	112.6 ± 36.9	128.2 ± 35.8	0.4920	0.6815	0.0127	0.0256
Glucose 6‐phosphate	156.8 ± 48.9	190.3 ± 53.8	286.0 ± 53.6	306.5 ± 75.3	0.6522	0.8896	0.0010	0.0029
Fructose 6‐phosphate	36.8 ± 12.5	43.0 ± 9.0	72.4 ± 13.2	77.1 ± 19.4	0.8065	0.9052	0.0002	0.0004
Fructose 1,6‐diphosphate	14.8 ± 3.9	44.5 ± 39.4	51.1 ± 38.8	56.2 ± 26.1	0.2721	0.9885	0.1343	0.8822
Lactic acid	8911.2 ± 1621.6	10198.3 ± 3194.7	11243.6 ± 2171.2	12217.0 ± 3370.2	0.7599	0.8781	0.3088	0.4309
Acetyl CoA_divalent	11.1 ± 4.7	19.1 ± 7.5	5.8 ± 2.6	3.5 ± 1.6	0.0179	0.7825	0.1695	<0.0001
Citric acid	58.1 ± 20.3	77.3 ± 29.0	84.0 ± 25.6	83.0 ± 36.6	0.5085	0.9999	0.2585	0.9741
Succinic acid	482.2 ± 144.7	337.9 ± 156.4	129.6 ± 34.8	195.2 ± 55.7	0.0243	0.5003	<0.0001	0.0261
Fumaric acid	263.6 ± 64.1	261.1 ± 72.6	245.0 ± 38.3	388.3 ± 104.7	0.9999	0.0032	0.9530	0.0091
Malic acid	726.3 ± 214.7	716.8 ± 265.7	660.4 ± 91.0	927.9 ± 231.0	0.9997	0.0615	0.9102	0.1794
3‐Hydroxybutyric acid	1193.8 ± 333.5	1535.3 ± 441.2	1390.5 ± 496.6	1279.9 ± 704.7	0.6112	0.9777	0.8910	0.7920
2‐Hydroxybutyric acid	141.3 ± 42.8	76.7 ± 38.8	94.7 ± 31.0	216.0 ± 67.7	0.0799	0.0005	0.2822	0.0001
Glycerol 3‐phosphate	1531.5 ± 238.5	1693.1 ± 398.1	1541.6 ± 325.9	1336.5 ± 427.8	0.7351	0.1375	>0.9999	0.1375

*Note*: Data are presented as mean ± standard deviation (SD), *n* = 8 per group. Statistical analysis was performed using two‐way ANOVA followed by Tukey's multiple comparison test.

Abbreviations: LHP, liquid high‐protein diet; LNC, liquid normal control diet; ND, nondiabetic.

### 3.6 Gene expression in the liver of ND and STZ‐D mice fed LNC or LHP

**FIGURE 3:**
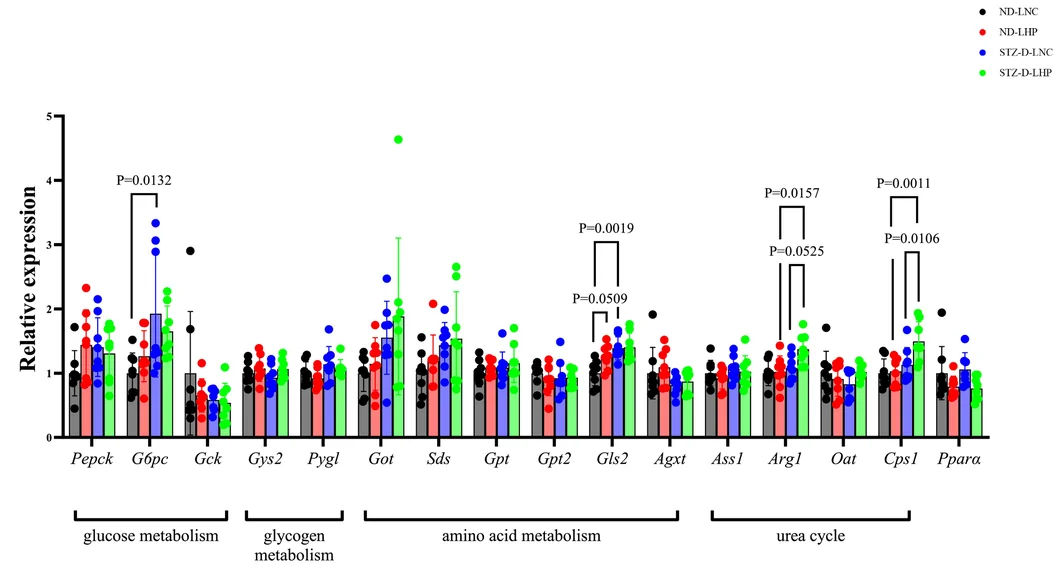
Hepatic mRNA expression levels of genes involved in glucose and amino acid metabolism; black bars, ND mice fed LNC; red bars, ND mice fed LHP; blue bars, STZ‐D mice fed LNC; green bars, STZ‐D mice fed LHP; Data are presented as mean ± standard deviation (SD), *n* = 8 per group. Statistical analysis was performed using two‐way ANOVA followed by Tukey's multiple comparison test. *p* Values are shown in the figure. *Agxt*, Alanine‐glyoxylate aminotransferase; *Arg1*, Arginase 1; *Ass1*, Argininosuccinate synthase 1; *Cps1*, Carbamoyl‐phosphate synthase 1; *G6pc*, Glucose‐6‐phosphatase, catalytic subunit; *Gck*, Glucokinase; *Gls2*, Glutaminase 2; *Got*, Glutamic‐oxaloacetic transaminase; *Gpt*, Glutamic‐pyruvic transaminase; *Gpt2*, Glutamic‐pyruvic transaminase 2; *Gys2*, Glycogen synthase 2; LHP, liquid high‐protein diet; LNC, liquid normal diet; mRNA, messenger Ribonucleic Acid; ND, nondiabetic mice; *Oat*, Ornithine aminotransferase; *Pepck*, Phosphoenolpyruvate carboxykinase; *Pparα*, Peroxisome proliferator‐activated receptor alpha; *Pygl*, Glycogen phosphorylase, liver form; *Sds*, Serine dehydratase; STZ‐D, diabetic model mice treated with STZ.

We next examined the expression levels of genes involved in glucose and amino acid metabolism in the liver. No significant differences in gene expression between LNC‐fed and LHP‐fed were observed except for *Cps1* in STZ‐D. In addition, a significant difference in expression levels was observed in a limited number of genes between ND and STZ‐D mice: *G6pc* and *Gls2* in the LNC‐fed group and *Arg1* and *Cps1* in the LHP group. These data suggest changes in gene expression play only a minor role in differential amino acid accumulation in the liver among the four groups (Figure 3).

### 3.7 Hepatic immunoblotting analysis in ND and STZ‐D mice fed LNC or LHP

**FIGURE 4:**
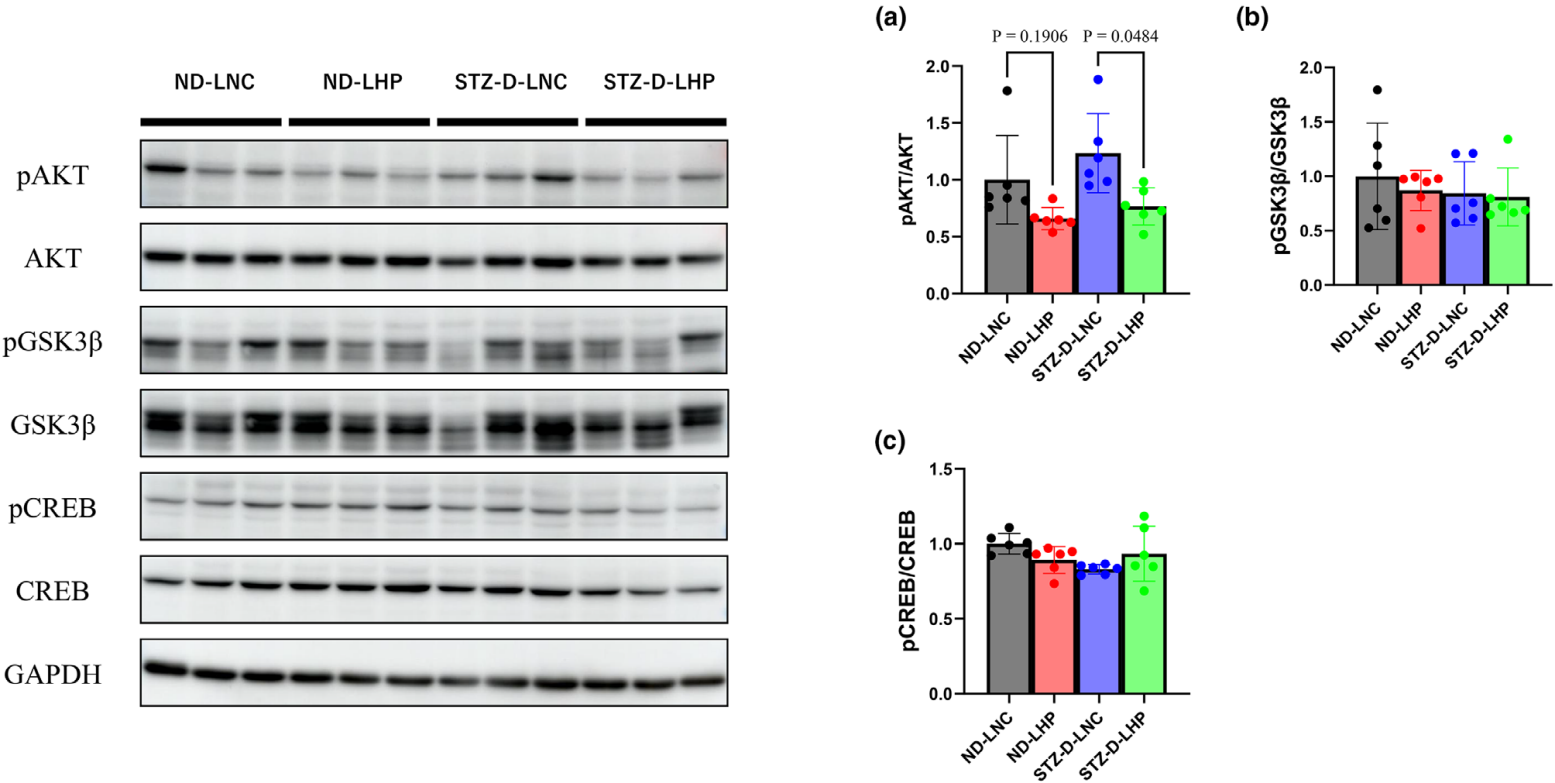
Hepatic immunoblotting analysis. Representative Western blot images and densitometric quantification of hepatic (a) phosphorylated AKT (p‐AKT)/total AKT, (b) phosphorylated GSK3β (p‐GSK3β)/total GSK3β, and (c) phosphorylated CREB (p‐CREB)/total CREB in ND and STZ‐D mice fed LNC or LHP. Phosphorylation levels were calculated as the ratio of phosphorylated to total protein and expressed relative to the mean value of the ND‐LNC group, which was set to 1.0. Representative GAPDH blots are shown as a loading control. Black bars, ND mice fed LNC; red bars, ND mice fed LHP; blue bars, STZ‐D mice fed LNC; green bars, STZ‐D mice fed LHP. Data are presented as mean ± standard deviation (SD), *n* = 6 per group. Statistical analysis was performed using two‐way ANOVA followed by Tukey's multiple‐comparison test. Exact *p* values are shown in the figure. AKT, protein kinase B; CREB, cAMP response element‐binding protein; GAPDH, glyceraldehyde‐3‐phosphate dehydrogenase; GSK3β, glycogen synthase kinase 3β; LHP, liquid high‐protein diet; LNC, liquid normal diet; ND, nondiabetic; STZ‐D, streptozotocin‐induced diabetic.

Finally, to evaluate insulin and glucagon signaling, we analyzed the phosphorylation levels of Akt, GSK3β, and CREB in mouse liver tissues at 120 min by western blotting. In ND mice, Akt phosphorylation tended to be decreased in the LHP group compared with the LNC group, although any difference was not statistically significant (Figure 4a). On the other hand, in STZ‐D mice, Akt phosphorylation was significantly lower in the LHP group than in the LNC group (Figure 4a). No significant differences were observed in GSK3β or CREB phosphorylation among the four groups (Figure 4b,c).

## 4 DISCUSSION

In the present study, we demonstrate that acute administration of a high‐protein diet induces rapid metabolic changes characterized by increased plasma glucagon and amino acids levels and differential hepatic amino acid metabolism between diabetic and nondiabetic states prior to detectable changes in gene expression in the liver. The STZ‐D mice exhibited attenuated insulin secretion in response to meal loading.

We first investigated the effects of an acute high‐protein diet on hormone secretion in both nondiabetic and diabetic mice. Glucose stimulates insulin and GIP secretion more effectively than protein (Ahrén, 2022; Elliott et al., 1993). In the present study, at 60 min after the meal load, plasma insulin levels were lower in the LHP‐fed ND mice than in the LNC‐fed ND mice, and plasma GIP levels were lower in the LHP group than in the LNC group. These findings are likely attributable to the lower carbohydrate content in the meal. Interestingly, despite a reduction in insulin secretion, the postprandial blood glucose levels after the meal load were lower in LHP‐fed STZ‐D mice than in LNC‐fed STZ‐D mice, whereas insulin secretion itself remained unchanged. This indicates that low carbohydrate content in meals plays a crucial role in improving postprandial blood glucose levels under conditions of impaired insulin secretion. Conversely, in LHP‐fed ND mice, plasma glucagon levels were significantly higher at 60 and 120 min than those in LNC‐fed ND mice, whereas in the STZ‐D mice, a significant increase was observed only at 120 min. This discrepancy may well be due to the enhanced glucagon secretion induced by hyperglycemia in STZ‐D mice, which may have partially masked the glucagon secretory response to LHP. On the other hand, plasma GIP levels were significantly lower at 60 min in the LHP group than in the LNC group, regardless of the presence of diabetes. This suggests that the phenomenon wherein protein stimulates less GIP secretion compared with glucose remains unaltered even in the diabetic state.

Despite evidence that chronic protein and sucrose intake modulate FGF21 in rodents (Laeger et al., 2014; Maekawa et al., 2017), the effects of acute administration remain largely unexplored. While acute intake of carbohydrate, such as glucose and fructose, elevates plasma FGF21 in human (Dushay et al., 2014; Samms et al., 2017), we found that plasma FGF21 levels were lower in ND‐LHP and STZ‐D‐LNC fed mice compared to those in ND‐LNC fed mice 2 h after administration of the diets. These results suggest that FGF21 secretion is acutely regulated by dietary protein levels and/or insulin action. Further studies are required to clarify how acute protein intake modulates FGF21 dynamics.

Glucagon and insulin coordinately regulate hepatic amino acid and glucose metabolism (James et al., 2017; Janah et al., 2019; Petersen & Shulman, 2018). While we previously reported that in mice fed a high‐protein diet for 7 days, plasma amino acids levels except for BCAAs do not differ from those in mice fed normal chow due to the glucagon‐mediated increase of hepatic expression of enzymes involved in amino acid metabolism (Ueno et al., 2022), the current study focused on the acute response to amino acids and/or glucagon on plasma amino acid concentrations in mice (Galsgaard et al., 2019; Galsgaard et al., 2020; Grøndahl et al., 2024; Kjeldsen et al., 2023; Sadri et al., 2017; Winther‐Sørensen et al., 2020). Since these responses occur instantaneously, a non‐transcriptional mechanism is assumed to be involved. Indeed, in the present study, no significant differences were observed in gene expression levels of enzymes in amino acid metabolism between LNC‐fed mice and LHP‐fed mice regardless of diabetes, 2 h after oral meal administration.

Hepatic glucose metabolism is coordinately regulated by insulin‐ and glucagon‐mediated signaling pathways. Insulin promotes glycogen synthesis through Akt‐dependent phosphorylation and inactivation of GSK3β, thereby relieving the inhibitory phosphorylation of glycogen synthase (Wang et al., 2022). In contrast, glucagon activates the cAMP–PKA–CREB signaling pathway, leading to the transcriptional induction of gluconeogenic genes and subsequently increasing hepatic glucose production (Janah et al., 2019). In the present study, Akt phosphorylation was significantly decreased in the LHP group compared to the LNC group, which is consistent with the lower liver glycogen content observed in the LHP group relative to the LNC group. However, phosphorylation of GSK3β, a key downstream effector of Akt in glycogen synthesis, showed no difference among the four groups. Furthermore, despite the elevated plasma glucagon levels in the LHP group and STZ‐D mice, CREB phosphorylation remained unchanged across the four groups. These discrepancies may be attributable to differences in temporal post‐feeding dynamics. Further investigation is required to analyze time‐course alterations in hormonal profiles, liver gene expression, and protein phosphorylation during feeding experiments.

Furthermore, although the IGR at 60 min was significantly decreased both in LHP‐fed ND mice and LNC‐fed STZ‐D mice compared with that in ND‐LNC, hepatic amino acid contents remained unchanged in LHP‐fed ND mice and were altered in only a subset of amino acids in LNC‐fed STZ‐D mice. These results indicate that amino acid metabolism is determined by multiple factors, which include nutrient availability, tissue‐specific demand, and the metabolic state, and not solely by the IGR.

Nevertheless, plasma amino acid profiles showed distinct changes: STZ‐D‐LNC mice exhibited lower levels of eight amino acids, including alanine, likely due to accelerated gluconeogenesis from amino acids (Korenfeld et al., 2021; Winther‐Sørensen et al., 2020), whereas all essential amino acids (EAAs) levels, except for phenylalanine in ND‐LHP mice, were higher in LHP‐fed mice than in LNC‐fed mice regardless of diabetic status. These results suggest that the acute postprandial amino acid excursion is primarily regulated by non‐transcriptional pathways—including the diversion of amino acids toward hepatic gluconeogenesis—and/or altered peripheral uptake, rather than immediate changes in hepatic enzymatic induction. The precise mechanisms remain to be fully elucidated.

Conventionally, insulin and glucagon are thought to act as opposing regulators of glycogen metabolism (Jiang & Zhang, 2003; Shah et al., 2000; Uehara et al., 2024; Wewer Albrechtsen et al., 2016), and diabetic models often exhibit reduced glycogen due to impaired insulin action (Del Prato et al., 1993; Gannon & Nuttall, 1997; Krssak et al., 2004). However, hepatic glycogen content was paradoxically higher in STZ‐D mice than in ND mice despite impaired insulin secretion and a lower insulin‐to‐glucagon ratio. Recently, it was reported that glucagon may also contribute to postprandial hepatic glycogen repletion after mix‐nutrient meal administration (Kejriwal et al., 2026). In addition, hyperglycemia and excess substrate availability promote hepatic glycogen accumulation even in diabetic states (Edgerton et al., 2004). The mechanism underlying hepatic glycogen accumulation in STZ‐D mice under refeeding conditions warrants further investigation.

While previous studies in obese diabetic models have attributed the increase in glycolytic intermediates to inefficient metabolic flux (van de Werve & Jeanrenaud, 1987), our findings in STZ‐D mice may suggest a distinct metabolic mechanism driven by overwhelming substrate availability. Following acute protein loading, we observed a marked accumulation of amino acids in the STZ‐D liver, together with elevated early gluconeogenic/glycolytic intermediates (glucose 6‐phosphate and fructose 6‐phosphate). These results may indicate that hepatic amino acid uptake exceeds the metabolic capacity of the liver following acute protein loading, leading to intrahepatic amino acid accumulation. The accumulated amino acids may serve as substrates for gluconeogenesis, together with increased expression of amino acid catabolic genes such as *Got1*, *Sds*, and *Gls2*, as well as gluconeogenic enzymes such as *Pepck* and *G6pc*. In addition, in LHP‐fed STZ‐D mice, hepatic contents of all six ketogenic amino acids were significantly higher than those in their ND counterparts. Conversely, among glucogenic amino acids, no significant differences were observed in cysteine, glutamic acid, glycine, nor histidine contents, whereas glutamine content was significantly decreased. These findings suggest that accelerated hepatic amino acid uptake is accompanied by stimulated gluconeogenesis in LHP‐fed STZ‐D mice. This metabolic shift likely prevents the accumulation of specific glucogenic amino acids in the liver, thereby shifting the metabolic flux toward gluconeogenesis.

Despite the higher hepatic glycogen content observed in STZ‐D mice, acute LHP intake resulted in significantly lower hepatic glycogen levels compared with LNC intake, particularly in STZ‐D mice. Because the LHP diet contained substantially less carbohydrate than the LNC diet, this reduction is likely attributable, at least in part, to decreased exogenous glucose availability.

This study has several limitations. First, we did not evaluate hepatic metabolism and gene expression under fasting condition or directly assess glycogen turnover. Second, we did not assess the absorption of nutrients from the intestinal tract. These issues should be addressed in future studies.

## 5 CONCLUSION

Acute intake of a high‐protein liquid diet induces rapid, non‐transcriptional metabolic changes, resulting in distinct hepatic amino acid accumulation patterns in diabetic mice. These findings highlight the importance of hormonal balance in modulating nutrient partitioning and suggest that metabolic sensitivity to protein loads is fundamentally altered in the diabetic state. In individuals with T1D, insulin secretion is severely impaired; protein‐rich meals may promote hyperglycemia by stimulating glucagon secretion (Dao et al., 2025). In contrast, our findings suggest that when residual insulin secretion is preserved, high‐protein, low‐carbohydrate feeding enhances hepatic amino acid uptake and glucogenesis without increasing postprandial blood glucose. The limited carbohydrate content may offset the glucose‐raising effect of glucagon‐stimulated glucogenesis and thereby attenuate postprandial glycemic excursions.

## AUTHOR CONTRIBUTIONS

**Hiroaki Sumioki:** Conceptualization; data curation; formal analysis; investigation; methodology; validation; visualization. **Eisuke Tomatsu:** Conceptualization; data curation; formal analysis; investigation; methodology; visualization. **Yusuke Seino:** Conceptualization; data curation; formal analysis; funding acquisition; investigation; methodology; project administration; validation; visualization. **Shihomi Hidaka:** Conceptualization; data curation; formal analysis; investigation; methodology; visualization. **Shinji Ueno:** Conceptualization; data curation; formal analysis; funding acquisition; investigation; methodology; validation. **Sayumi Kanie:** Investigation. **Koki Nishida:** Investigation. **Takuya Haraguchi:** Conceptualization; methodology. **Naoya Murao:** Conceptualization; methodology. **Haruki Fujisawa:** formal analysis; Writing – review and editing. **Takeshi Takayanagi:** Data curation; formal analysis. **Yoshihisa Sugimura:** formal analysis; Writing – review and editing. **Yoshitaka Hayashi:** Conceptualization; methodology; project administration. **Atsushi Suzuki:** Supervision.

## FUNDING INFORMATION

This study was supported by Grants‐in‐Aid for Scientific Research from the Japan Society for the Promotion of Science to Yu.S. (21K11608 and 24K14638), grants from the Japan Association for Diabetes Education and Care, 23‐2558 and 23‐2667 (to Yu.S. and S.U.), and a research grant from Fujita Health University.

## CONFLICT OF INTEREST STATEMENT

The authors declare that they have no conflict of interest.

## ETHICS STATEMENT

All animal experiments and research protocols were approved by the Ethics Committee of Fujita Health University (AP18002; approval date: 14 May 2018).

## DECLARATION OF GENERATIVE AI AND AI‐ASSISTED TECHNOLOGIES IN THE WRITING PROCESS

The authors used Gemini (Gemini‐3.5 Thinking, Open AI; used between March 2026 and August 2026) and ChatGPT (GPT‐5.5 Thinking, OpenAI; used between March 2026 and August 2026) to enhance the English phrasing of this manuscript.

## Supporting information


Figures S1–S2.

## Data Availability

The data used to support the findings of this study are available from Yu.S. (Yusuke Seino) upon request. All metabolomics data for the liver are available in Metabobank under accession number MTBKS285 (https://ddbj.nig.ac.jp/public/metabobank/study/).
