# Supplementary material for: Acute high‐protein feeding induces hepatic amino acid accumulation in diabetic male mice prior to transcriptional adaptation
Source: Physiol Rep. 2026 Sep 25;14(18):e71117. doi: 10.14814/phy2.71117 (PMC13615171; doi:10.14814/phy2.71117)

Supplemental Figure. 1

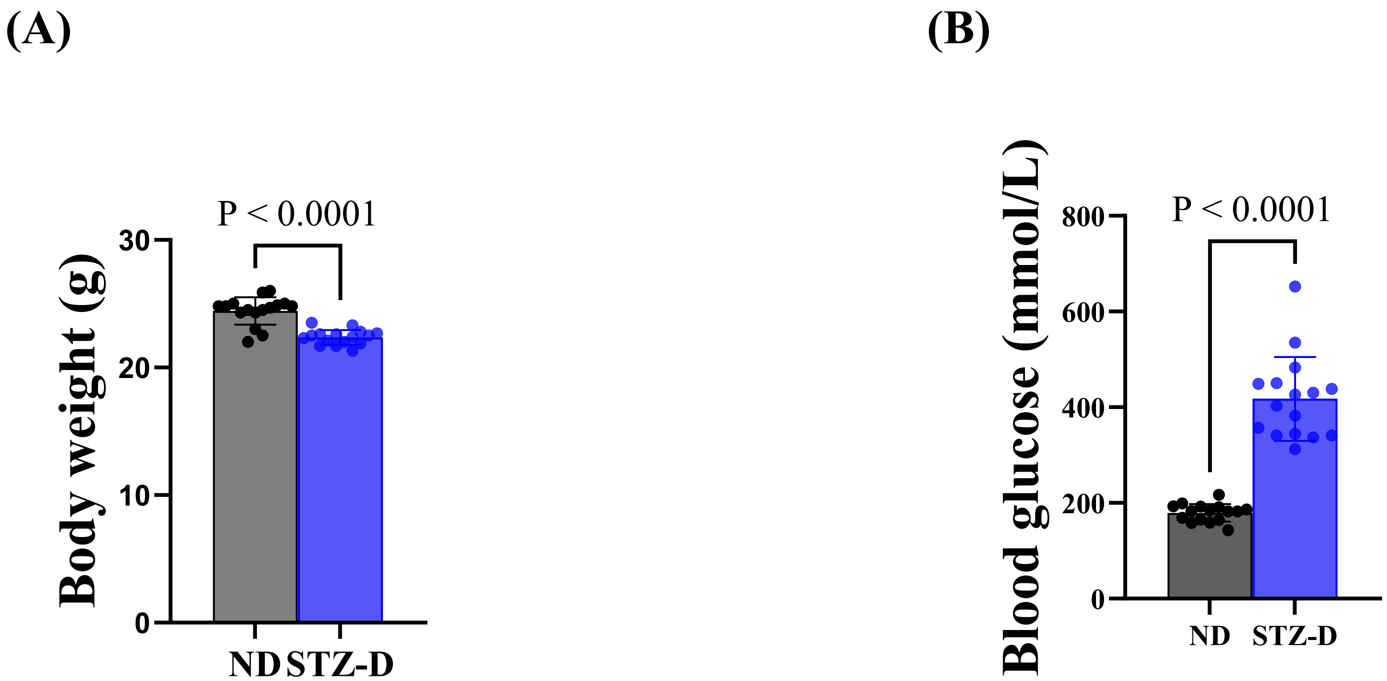

Supplement: Supplementary file 1 [file PHY2-14-e71117-s001.zip › PHYSREP-2026-05-443-s02.pdf]
