# Supplementary material for: Acute high‐protein feeding induces hepatic amino acid accumulation in diabetic male mice prior to transcriptional adaptation
Source: Physiol Rep. 2026 Sep 25;14(18):e71117. doi: 10.14814/phy2.71117 (PMC13615171; doi:10.14814/phy2.71117)
Supplement: Supplementary file 1 [file PHY2-14-e71117-s001.zip › Captions.docx]

**Supplemental Figure 1. Body weight and blood glucose levels in ND and STZ-D mice before initiating a 16-h fasting period.** (A) Body weight and (B) blood glucose levels in ND and STZ-D mice. Gray bars, ND mice; blue bars, STZ-D mice. Data are presented as mean ± standard deviation (SD), *n* = 16 per group. Statistical analysis was performed using an unpaired two-tailed Welch’s *t*-test. Exact *P* values are shown in the figure. Abbreviations: ND, non-diabetic; STZ, streptozotocin; STZ-D, streptozotocin-induced diabetic.

**Supplemental Figure 2. Uncropped Western blot images corresponding to Figure 4.**
Uncropped Western blot images of hepatic phosphorylated AKT (p-AKT; Ser473), total AKT, phosphorylated GSK3β (p-GSK3β; Ser9), total GSK3β, phosphorylated CREB (p-CREB; Ser133), total CREB, and GAPDH in ND and STZ-D mice fed LNC or LHP. For each protein, the left and right images show biological replicates 1–3 and 4–6, respectively, for the ND-LNC, ND-LHP, STZ-D-LNC, and STZ-D-LHP groups. Molecular weight markers and the expected molecular weights of the target proteins are indicated. The corresponding target-band regions were cropped and used to prepare the representative Western blot images shown in Figure 4. GAPDH was used as a loading control. Abbreviations: AKT, protein kinase B; CREB, cAMP response element-binding protein; GAPDH, glyceraldehyde-3-phosphate dehydrogenase; GSK3β, glycogen synthase kinase 3β; LHP, liquid high-protein diet; LNC, liquid normal diet; ND, non-diabetic; STZ-D, streptozotocin-induced diabetic.
